# Malaria case management in Papua New Guinea prior to the introduction of a revised treatment protocol

**DOI:** 10.1186/1475-2875-11-157

**Published:** 2012-05-07

**Authors:** Justin Pulford, Ivo Mueller, Peter M Siba, Manuel W Hetzel

**Affiliations:** 1 Papua New Guinea Institute of Medical Research, PO Box 60, Goroka, EHP 441, Papua New Guinea; 2 The University of Queensland, School of Population Health, Herston, Qld, 4006, Australia; 3 Barcelona Centre for International Health Research, Barcelona, Spain; 4 Walter and Eliza Hall Institute of Medical Research, Melbourne, Australia

## Abstract

**Background:**

This study aimed to document malaria case management practices in Papua New Guinea prior to the introduction of a revised national malaria treatment protocol. The revised protocol stipulates routine testing of malaria infection by rapid diagnostic test or microscopy, anti-malarial prescription to test positive cases only, and the introduction of a new artemisinin-based first-line anti-malarial. Findings presented in this paper primarily focus on diagnostic, prescription and treatment counselling practices.

**Methods:**

In a national cross-sectional survey of 79 randomly selected health facilities, data were collected via non-participant observation of the clinical case management of patients presenting with fever or a recent history of fever. Data were recorded on a structured clinical observation instrument.

**Results:**

Overall, 15% of observed fever patients (n = 468) were tested for malaria infection by rapid diagnostic test and a further 3.6% were tested via microscopy. An anti-malarial prescription was made in 96.4% (451/468) of cases, including 100% (17/17) of test positive cases and 82% (41/50) of test negative cases. In all, 79.8% of anti-malarial prescriptions conformed to the treatment protocol current at the time of data collection. The purpose of the prescribed medication was explained to patients in 63.4% of cases, dosage/regimen instructions were provided in 75.7% of cases and the possibility of adverse effects and what they might look like were discussed in only 1.1% of cases.

**Conclusion:**

The revised national malaria treatment protocol will require a substantial change in current clinical practice if it is to be correctly implemented and adhered to. Areas that will require the most change include the shift from presumptive to RDT/microscopy confirmed diagnosis, prescribing (or rather non-prescribing) of anti-malarials to patients who test negative for malaria infection, and the provision of thorough treatment counselling. A comprehensive clinician support programme, possibly inclusive of ‘booster’ training opportunities and regular clinical supervision will be needed to support the change.

## Background

Malaria is a major public health problem in Papua New Guinea (PNG). In 2009, out of a population of close to seven million people, official statistics report 1,431,395 suspected malaria cases, 22,896 inpatient malaria cases and 604 malaria attributable deaths [1]. Malaria endemicity in PNG ranges from holo-endemic transmission in coastal and island regions to unstable transmission with localized epidemics at altitudes between 1,300 to 1,600 m to no transmission above 1,700 m [2]. In total, over 90% of the population are considered at risk of malaria infection and over 50% live in areas with potentially high transmission below 1,200 m [1,2]. Anti-malarial medicines are available via the retail sector in PNG and traditional therapy continues to be utilized [3], although evidence suggests that malaria treatment is most often sought from government and mission-run health facilities [4,5]. This health facility network comprises four levels of service provision including hospital, health centre, health sub-centre, and aid-post. The hospital is the largest unit of health service provision in terms of the number and range of clinical staff employed and the number and range of clinical services provided. The aid-post is the smallest unit of health service provision and is typically staffed by a single health worker with two years clinical training. In theory, all cases of uncomplicated malaria are treatable at the aid-post level with severe cases referred to a health centre or hospital.

Malaria case management across this health facility network is standardized against a national treatment protocol. The protocol current at the time of this study (from here on referred to as the ‘former’ protocol) stipulated the provision of anti-malarials to all children with fever and, where microscopy is not available, presumptive diagnosis in febrile adults [6,7]. First line treatments included: amodiaquine plus sulphadoxine-pyrimethamine (SP) or chloroquine plus SP for the treatment of uncomplicated malaria and artemether injection plus artesunate tablets plus SP or, if artemether and artesunate we are unavailable, quinine injection plus quinine tablets plus SP, for the treatment of severe malaria. Given widespread resistance to these first line anti-malarials in PNG [8-10], and in line with international recommendations [11], a revised national malaria treatment protocol was introduced in late 2011. The revised protocol stipulates that all fever or suspected malaria cases be tested for malaria infection by microscopy or rapid diagnostic test (RDT) with the possible exception of children under five years of age with signs of severe illness, introduces artemether-lumefantrine (AL) as the new first-line treatment for uncomplicated falciparum malaria, AL plus primaquine as the new first-line treatment for uncomplicated vivax malaria and artesunate injection plus AL for the first-line treatment of severe malaria, whether falciparum or vivax [12]. These guidelines emphasize the importance of patient counselling particularly in order to ensure patient adherence to the treatment regimen and to minimize the risk of development of drug resistance. In addition, potential side effects associated with primaquine in G6PD deficient individuals may warrant close supervision of patients on primaquine treatment.

The introduction of a test-and-treat policy is a major change to the routine of health care workers who are used to treating most malaria cases presumptively based on a clinical diagnosis. The experience of other malarious countries that have introduced similar revisions to their respective malaria treatment protocols suggests health worker adherence may be problematic. In Kenya, for example, three years after the implementation of a new treatment protocol stipulating routine microscopy or RDT testing of all adult fever cases and the prescription of AL to test positive cases, testing rates in health facilities with RDT or microscopy available did not exceed 54% and nearly a third of test negative cases were prescribed AL [13]. Similar or worse rates of health worker adherence to malaria case management guidelines have been reported elsewhere [14-16]. Attempts to understand why health workers may be reluctant to adhere to current best practice malaria case management guidelines suggest a possible lack of confidence in RDT results, intentional rationing of ACT prescription due to fear of possible stock-outs or the perceived cost, a desire to utilize (rather than waste) existing stocks of former first-line anti-malarials, and inappropriate and/or inadequate training and follow-up support [17,18].

Whatever the barriers to a quick and full adherence to a new treatment protocol, it is reasonable to assume that the greater the change in clinical practice required, the greater the potential obstacles are likely to be. At the time of this study it was largely unknown how substantial a shift in malaria case management practice the revised guidelines would require of PNG health workers. Malaria microscopy and RDT kits were available in PNG prior to the new protocol, but the extent of their availability and subsequent use had not been widely investigated. Equally under-investigated were contemporary anti-malarial prescription and patient counselling practices. Even the quality of presumptive diagnostic practices, the primary basis for anti-malarial prescription under the former protocol and a possible feature of the new protocol for fever patients less than five years of age, had not been previously examined. Accordingly, this study aimed to document malaria case management practices in PNG prior to the introduction of the new malaria treatment protocol. It was anticipated that the resulting findings would highlight potential barriers to the successful implementation of the new protocol and would serve as a baseline from which to measure the degree of change in clinical practice that occurs following its introduction. Specific research questions included: What percentage of fever cases are tested for malaria infection by microscopy or RDT? What percentage of fever cases are prescribed an anti-malarial in accordance with the current national malaria treatment protocol? What percentage of test negative fever cases are prescribed an anti-malarial? What percentage of fever cases prescribed an anti-malarial are: advised on the purpose of the medication, dosage/regimen instructions, dietary considerations, or possible adverse effects; instructed to return for further treatment if their symptoms persist or deteriorate; given malaria prevention advice.

## Methods

### Study sites

PNG comprises 20 provinces split into four regions known as Momose, Southern, Islands, and Highlands. This national cross sectional survey was carried out in areas with endemic or potentially epidemic malaria. The study sample consisted of two health centres or health sub-centres (collectively referred to as health centres in this paper) and up to four aid posts selected from each province using a simple random sampling procedure. The sampling frame included all health centres operational in March 2010 inclusive of government and mission administered health facilities (N = 689). Aid posts were randomly selected on site at participating (i.e. randomly selected and consenting) health centres. The sampling frame for aid posts was all operational aid posts under the supervision of the health centre at the time of survey. Hospitals were excluded from the sampling frame as they are few in number and serve only a minority of the PNG population. Conversely, health centres and aid posts are widely available across the country and are the main providers of primary care.

### Survey procedure

The study was carried out from June to November 2010 and was conducted by three trained field teams, each comprising three members, working simultaneously at different sites. The training programme for field staff spanned 10 days and consisted of lectures on the project background, malaria facts and effects, survey methodology, and intensive instruction and practice on the survey instruments. Members of each survey team spent between three to five days at each participating health centre and up to one day at each participating aid post. Four survey instruments were completed at each health facility, although this paper only reports data obtained from the non-participant observation of malaria case management (described below). The field team members sought to observe the case management of all fever cases presenting to each health facility for the duration of their visit. Prior to any health facility visit, the respective provincial and district health authorities were informed of the study objectives, sites, and timetable. The provincial health authority was also asked to commission a health officer to accompany the field team. Oral informed consent was sought from the officer in charge at all participating health facilities and from all participating clinicians and patients prior to clinical observation. The study was approved and granted ethical clearance by the Medical Research Advisory Committee of PNG (MRAC No. 10.12; 26 Feb 2010).

### Survey instrument

The survey instrument was designed to record observed features of the clinical case management of patients presenting with fever or a recent history of fever. The instrument was divided into discrete sections including consultation and diagnosis, prescription and treatment counselling. The content of each section was informed with input from experienced medical- and medical research- professionals. The instrument was completed by a trained field team member who would silently observe the management of fever patients from the point of initial contact with a health professional until service exit or admission onto a treatment ward. During the course of this observation, the field team member recorded on the structured clinical observation instrument whether specified actions did or did not occur and recorded the content of specific actions (e.g. whether an RDT was conducted and, if yes, what was the outcome?). Eligible patients were identified upon first contact with a health worker or, if circumstances allowed, by screening in the waiting area prior to first contact with a health worker.

### Data analysis

All data were double entered into DMSys version 5.1 (Sigma Soft International). Intercooled Stata version 9 was used for descriptive data analysis and for calculating 95% confidence intervals (CIs). Odds ratios with their corresponding 95% CI were calculated to assess the association between selected patient, clinician and health facility factors and receiving (or not) a diagnostic test for malaria infection. The calculation of all CIs was adjusted for possible clustering at the health facility level.

## Results

### Sample

A total of 605 clinical observations were completed with patients presenting with fever or a recent history of fever during the survey period. Patients who had been treated for fever or malaria infection within 14 days prior to interview were subsequently removed from analysis to ensure the findings better represented initial malaria case management practice. This restriction resulted in a final sample of 468 clinical observations obtained from 54 health facilities. Table 1 presents sex and age characteristics of the observed fever patients by region.

**Table 1:** Sex and age of the clinical observation sample by region (n = 468)

**Characteristic**		**Region**				**Overall**
		Southern	Highlands	Momase	Islands	
Female n (%)		64 (51)	50 (46)	56 (46)	51 (47)	221 (47)
Age n (%)	0–4 yrs	60 (48)	48 (46)	58 (48)	49 (45)	215 (47)
	5–15 yrs	31 (24)	14 (14)	30 (25)	35 (33)	110 (24)
	16+ yrs	35 (28)	41 (40)	32 (27)	24 (22)	132 (29)

### Diagnosis

Table 2 lists the percentage of fever patients observed discussing a specified topic or receiving a specified procedure during the initial clinical consultation. As shown, the topics most likely to be discussed during the clinical consultation were the presence or recent experience of fever (96.5% of cases), the duration of reported symptoms (90.5% of cases) and the presence or recent experience of cough (78.8% of cases). The topics least likely to be discussed included pregnancy status (only 16% of females aged between 15 and 40 years were questioned about it), current use of any medication (38.9% of cases) and the presence or recent experience of chills (39.2% of cases). The three most frequently observed procedures included the examination of a patient’s health card (92% of cases), measuring body temperature (86.8% of cases) and measuring body weight (76.6% of cases). The other specified procedures were observed in fewer than 17% of cases. In only 15% of cases was an RDT carried out and in only 3.6% of cases was a blood smear taken. The average number of specified topics discussed and specified procedures performed per observation was six out of a possible total of nine (range 0–9; pregnancy status was excluded from this calculation) and three out of a possible total of eight (range 0–7; RDT and blood smear were collapsed into a single ‘diagnostic test’ category for this calculation), respectively.

**Table 2:** Percentage of fever/suspected malaria patients observed discussing a specified topic or receiving a specified procedure during initial clinical consultation

**Topic of Discussion/Performed Procedure**		**n**^a^	**Occurrence (%)**	**95% CI**
Discussion	Current use of any medication	453	38.9	(30.9, 47.4)
	Concurrent illness/existing condition	452	74.3	(64.7, 82.1)
	Pregnancy status^b^	51	15.7	(6.5, 33.3)
	Presence/recent experience of fever	457	96.5	(91.8, 98.5)
	Presence/recent experience of cough	453	78.8	(73.2, 83.5)
	Presence/recent experience of head/body ache/pain	449	56.6	(48.8, 64.0)
	Presence/recent experience of nausea/vomiting	453	63.4	(55.4, 70.6)
	Presence/recent experience of diarrhoea	453	60.5	(53.2, 67.3)
	Presence/recent experience of chills	444	39.2	(30.8, 48.3)
	Duration of current symptoms	455	90.5	(86.4, 93.5)
Procedure	Health card examination	465	92.0	(87.5, 95.0)
	Body temperature measurement	463	86.8	(81.1, 90.4)
	Body weight measurement	462	76.6	(68.4, 83.2)
	Blood pressure measurement	455	4.6	(2.8, 7.5)
	Abdomen palpation	461	16.9	(10.8, 25.6)
	Eyes examination	460	16.3	(11.5, 22.6)
	Palms examination	459	3.3	(1.5, 7.2)
	Blood slide taken or referral made	468	3.6	(1.2, 10.6)
	RDT conducted or referral made	468	15.0	(7.3, 28.2)

a. Each specified topic/procedure was scored ‘observed’, ‘not observed’ or ‘don’t know’. All ‘don’t know’ responses were excluded from the analyses, hence the variation in reported numbers. b. Sample limited to females 15–40 years of age.

Twelve (22%) of the 54 health facilities in which the 468 fever patients were collectively observed had unexpired RDT in stock or an operational microscope available at the time of the survey. Overall, 137 (29.3%) of the 468 fever patients were observed at one of these 12 health facilities. Table 3 presents the percentage of these 137 patients tested for malaria infection by RDT, by blood slide or by RDT or blood slide (as some overlap was observed). The percentage of patients tested by RDT or bloodslide in these health facilities varied by age group; 30.4% (17/56) of patients aged less than five years were tested for malaria infection compared to 50% (14/28) of patients aged between five and fifteen years and 47.9% (23/48) of patients aged 16 years or older.

**Table 3:** Percentage of observed fever/suspected malaria patients tested for malaria infection by blood slide, RDT or either blood slide or RDT at health facilities in which RDT and/or microscopy were available (n = 137)

**Test Type(s)**	**% Tested**	**CI (95%)**
Blood slide	10.9	(3.2, 31.6)
RDT	35.8	(17.1, 60.0)
Blood slide or RDT	40.9	(21.1, 64.1)

Factors potentially predictive of being tested for malaria infection by RDT or microscopy were examined by logistic regression among the 137 patients who presented to one of the 12 health facilities with relevant diagnostic capacity. The outcome variable was whether a patient had been tested for malaria infection by RDT or microscopy (yes/no). Predictor variables included patient age, patient sex, location of health facility, clinician qualification, and clinician experience. Results are presented in Table 4. As can be seen, patients were more likely to be tested for malaria infection by RDT or microscopy if they were five years or older, treated by a health worker with more than 20 years clinical experience or if they attended a health facility in the Highlands region.

**Table 4:** Patient, clinician and health facility factors associated with receiving a diagnostic test for malaria infection in Papua New Guinea

**Factors**	**Diagnostic Status**			**OR (95% CI)**	***p***
	Test	No test		
Patient Age				
Less than 5 years of age	17	39	1.00	
5+ years of age	37	39	2.7 (1.1, 6.7)	0.032
Patient Sex				
Female	29	37	1.00	
Male	27	44	0.7 (0.3, 1.7)	0.440
Health Worker Qualification				
Community Health Worker	21	35	1.00	
HEO/nursing officer	32	42	1.4 (0.5, 3.5)	0.519
Clinical Experience				
Less than 20 years experience	26	33	1.00	
20+ years experience	26	17	2.5 (1.0, 6.3)	0.050
Health Facility Location				
Highlands (less malarious)	27	20	1.00	
Southern, Momase, Islands (more malarious)	29	61	0.2 (0.1, 0.7)	0.006

### Prescription

Overall, 96.4% (451/468) of the observed fever patients were provided with an anti-malarial prescription. The type of anti-malarial(s) prescribed was recorded in 98% (440/451) of cases. Of the 440 anti-malarial prescriptions recorded, 79.8% (351/440) conformed to one or another of the treatment recommendations current at the time of data collection. This included prescriptions of amodiaquine plus SP (n = 167) or chloroquine plus SP (n = 148), quinine plus SP (n = 11), artemether or artesunate plus SP (n = 7), and chloroquine plus SP plus primaquine which is recommended for the treatment of vivax malaria in adult patients (n = 18). Of the remaining prescriptions (89/440), 68 were monotherapies (amodiaquine n = 27, chloroquine n = 23, artemether n = 14, quinine n = 3, SP n = 1) or non-recommended anti-malarial combinations (artemether + primaquine n = 8m SP + primaquine n = 3, amodiaquine + primaquine n = 3, amodiaquine + chloroquine n = 2, amodiaquine + artemether n = 1, chloroquine + primaquine n = 1, chloroquine + quinine n = 1, chloroquine + SP + artemether n = 1, and chloroquine + SP + doxycycline n = 1).

Of the 351 patients provided a recommended anti-malarial prescription, 39.3% (138/351) were required to return to their respective health facility to complete the prescription (i.e. ‘take away’ anti-malarial medication was not provided), 57.3% 201/351 were provided a full prescription to take home, and in 3.4% (12/351) of cases the relevant information was not recorded. Of the 201 patients provided a recommended anti-malarial prescription in take away form, the correct dosage for the respective regimen was provided in 75.6% (152/201) of cases, an incorrect dosage was provided in 15.9% (32/201) of cases, and in 8.5% (17/201) of cases a determination as to the correctness (or otherwise) of the provided medication dosage could not be made.

Factors potentially predictive of being prescribed a recommended anti-malarial were examined by logistic regression among the 440 patients prescribed an anti-malarial and for whom this prescription was recorded. The outcome variable was whether a patient received a recommended anti-malarial combination (yes/no), excluding dosage considerations. The predictor variables were those previously described in Table 4. Results are presented in Table 5. As can be seen, patients were more likely to be prescribed a recommended anti-malarial if they were treated by a health worker with less than 20 years’ clinical experience or if they attended a health facility in an area other than the Highlands region.

**Table 5:** Patient, clinician and health facility factors associated with receiving a recommended anti-malarial prescription for malaria infection in Papua New Guinea

**Factors**	**Recommended Prescription**		**OR (95% CI)**	***p***
	Yes	No		
Patient age				
Less than 5 years of age	158	46	1.00	
5+ years of age	184	42	1.3 (0.7, 2.2)	0.397
Patient sex				
Female	168	45	1.00	
Male	182	45	1.1 (0.6, 1.9)	0.739
Health Worker Qualification				
Community Health Worker	195	57	1.00	
HEO/nursing officer	134	29	1.5 (0.8, 2.8)	0.192
Clinical Experience				
Less than 20 years experience	144	24	1.00	
20+ years experience	132	50	0.4 (0.2, 0.7)	0.002
Health Facility Location				
Highlands (less malarious)	69	28	1.00	
Southern, Momase, Islands (more malarious)	282	62	2.6 (1.3, 5.1)	0.004

Of the 78 patients who were tested by RDT or blood slide, the test result was recorded in 69 (86%) cases. Of these 69 patients, 72% (50/69) tested negative for malaria infection, 15% (17/69) positive and the test result was invalid in 3% (2/69) of cases. An anti-malarial was subsequently prescribed to 84% (58/69) of these patients, including 82% (41/50) of patients who tested negative for malaria infection and 100% (17/17) of test positive cases. The blood slide or RDT result was available at the time of anti-malarial prescription in 83% (57/69) of these cases including 39 out of the 41 patients who tested negative for malaria infection and were subsequently prescribed anti-malarials.

### Counselling

Table 6 displays the proportion of fever patients observed to have been provided with each of six different ‘types’ of clinical instruction by their respective clinician(s). The sample was restricted to patients who had been prescribed anti-malarial medication. As shown, the dosage instructions and the purpose of the medication supplied were explained in the majority of cases (75.7% and 63.4%, respectively). Treatment re-engagement instructions were the next most frequently provided at 27.7%. Malaria prevention advice, dietary instructions and the possible side effects of medication supplied were provided in 10.3%, 6.2% and 1.1% of cases, respectively. The duration of clinical consultation, from first to last contact with a health worker/s (inclusive of any waiting time between first and last contact), was recorded in 93% (437/468) of cases. The mean consultation time was 19.4 minutes (SD 15.9).

**Table 6:** Observed provision of instructions

**Instruction**	**n**^a^	**Provided (%)**	**(95% CI)**
Purpose of medication	435	63.4	(53.7, 72.0)
Dosage/regimen	441	75.7	(67.0, 82.5)
Dietary	449	6.2	(3.5, 10.8)
Possible adverse effects	449	1.1	(0.5, 2.6)
Health facility re-engagement^b^	447	27.7	(19.7, 37.4)
Prevention advice	448	10.3	(6.4, 16.1)

a. Each instruction was scored ‘provided’, ‘not provided’ or ‘don’t know’. All ‘don’t know’ responses were excluded from the analyses, hence the variation in reported numbers.

b. In which patients are advised to return to the health facility if current symptoms persist or deteriorate.

## Discussion

Findings from this study indicate that microscopy or RDT confirmed malaria diagnosis was the exception in PNG under the former treatment protocol, collectively occurring in less than 20% of observed malaria case management consultations. Microscopy or RDT was only available in 22% of the surveyed health facilities, which clearly limited their use, although even in those facilities with these diagnostic tools available only 40% of fever patients were tested. Patients older than five years of age were more likely to be tested for malaria infection relative to younger patients, although still fewer than 50% of adult (16 years +) patients were tested in facilities with the resources to do so. The study findings also suggest that clinical diagnosis was often a far from exhaustive process. Questions that could reasonably be expected to be a mandatory component of a thorough clinical assessment were often not asked and procedures such as palpating the abdomen or examining the patients’ eyes or palms were rarely conducted. Thus, it would appear that most malaria diagnoses in PNG were made presumptively, simply on the basis of the presence of fever without a thorough clinical examination and use of diagnostic tests. Anti-malarial provision to fever patients was near universal with 96.4% of the observed fever cases receiving a prescription, including the 41/50 patients who tested negative for malaria infection by RDT or blood slide.

These findings are of concern from a treatment perspective as evidence suggests many (and in some cases most) fever patients in PNG, when tested by microscopy or RDT, do not have malaria infection [19]. Numerous patients, therefore, are likely to be receiving anti-malarials unnecessarily and, as a result, may have experienced a delay in appropriate diagnosis and treatment response. The consequences of malaria misdiagnosis have not been well examined in PNG, although international evidence suggests it may contribute to ongoing ill health and economic hardship, especially amongst the poorest members of a community [20,21]. Malaria misdiagnosis further contributes to the development of parasite resistance to anti-malarial drugs, a significant problem in PNG at present [22] and an issue of global concern with respect to containing parasite resistance to the new artemisinin-based anti-malarial drugs [23,24]. A malaria misdiagnosis also incurs economic costs in the form of unnecessary medication prescription, an issue of concern in a developing country.

These findings highlight the substantial changes that the revised PNG national malaria treatment protocol will require in terms of malaria case management practice. In particular, the proposed shift to routine testing of all fever patients by RDT or microscopy and the prescription of anti-malarials to test positive cases only are likely to challenge currently entrenched practice. The emphasis on thorough patient counselling in the new treatment protocol is also likely to take some adjusting to given the practices observed in this study. For example, the purpose of the prescribed medication was not explained to patients in 36% of cases, dosage/regimen instructions were not provided in 25% of cases, the possibility of adverse effects and what they might look like were virtually never discussed, and instructions on when to return to the health facility (if needed) were only provided in 28% of cases. On a more positive note, 79% of prescriptions conformed to current treatment guidelines and the prescription of mono-therapies was relatively rare (68/440 prescriptions). These latter findings indicate most clinicians are generally aware of and comply with recommended prescription practices and may continue to do so with the introduction of a revised protocol. Conversely, these findings also indicate that outdated mono-therapies were provided in over 15% of cases and the treatment guidelines were not followed in 21% of cases. Thus, full adherence to the new protocol in the short- to mid-term is probably unrealistic if the same has not been achieved with the former, familiar protocol.

Realistically, the successful transition from the former to the revised national malaria treatment protocol, in terms of health worker compliance, is likely to take an extended period of time; all the more so given the degree of change in clinical practice that will be required. The experience from other developing countries that have previously revised their national treatment protocols in a manner consistent with that proposed in PNG is instructive in this regard. In Kenya, in the first year post-implementation of an ACT-based national malaria treatment protocol, ACT was prescribed to fewer than 30% of fever patients [25]. Four years later the percentage of fever patients prescribed ACT in Kenya had risen to over 60% [13]. Consistent with the Kenyan experience, a recent review of health worker prescribing practices identified that compliance with ACT prescribing guidelines increases the longer guidelines have been in place [26]. However, it was notable that even in health facilities in which ACT was in stock, the prescription of ACT to fever cases was below 70% in every study included in the review and typically below 50% if the study was conducted shortly after implementation of the ACT-based guidelines. Findings pertaining to RDT use depict a similar scenario. Health worker compliance with the use of RDT kits to test for malaria infection has been low [27], although not always [28], in the period immediately following their introduction. A high level of health worker compliance with RDT results also appears difficult to achieve with many studies reporting anti-malarial prescription to between 30 to 50+ % of test negative cases [27-29], even when the testing programme has been in place for some years [13].

What these studies collectively suggest is that achieving a high level of health worker compliance to a revised RDT/ACT-based malaria treatment protocol takes a number of years. A high proportion, possibly a third or more, of test negative cases are still likely to be prescribed anti-malarials some time after the introduction of RDT kits and it is unlikely that more than 70–80% of test positive cases will be prescribed the correct ACT even two to three years post-implementation of a revised protocol. Progress in PNG is unlikely to be any faster given the level of change in malaria case management practice that will be required. The potential barriers to health worker compliance with malaria treatment protocols are multiple, ranging from supply problems to peer/patient pressures to insufficient and/or inappropriate training/support to mistrust in the new medicines or diagnostic resources [17,18,30,31]. A small number of studies have sought to identify interventions that might usefully improve adherence to malaria treatment guidelines, although often with limited success. For example, the impact of a three-day, in-service training and the provision of various resources and job aids on malaria case management were modest at best in a pre-/post-intervention study conducted in Kenya [32]. The authors subsequently concluded that one-off training interventions, even when supported by training materials and job aids, are unlikely to be effective if follow-up support and supervision is not provided. Reflecting the benefit of regular longer-term support, a recent study demonstrated a substantial improvement in health worker adherence to malaria treatment guidelines via the provision of regular text message reminders [33]. Ten discrete text messages, each describing a recommended malaria case management practice, were variously sent to participating health workers personal mobile phones twice a day, five days a week over a six-month period. A 24.5% improvement in malaria case management practice (based on adherence to national treatment guidelines) was subsequently observed in the intervention group *versus* the control group six months post intervention. Despite this promising result, there remain few interventions that have been reliably demonstrated to improve malaria case management practice [34] and although regular supervision and follow-up support (via any medium) may improve performance, complete or even substantial adherence to a revised malaria treatment protocol will still likely require an extended time period.

The study presented in this paper was not without limitations. The final sample size, and especially the number of aid posts included in the sample, was lower than anticipated. This was largely due to the inaccessibility of aid posts or, more frequently, the absence of any functional aid post to survey. The study was conducted during a period of low malaria transmission (June-November, 2010) in those provinces with seasonal variation. Thus, the number of malaria patients presenting to health facilities and the subsequent pressure on staff and resources (e.g. RDT kits, anti-malarial medication) may have been lower during the survey period as opposed to peak transmission periods. Clinical observations may also have been subject to some form of bias given that participating clinicians were aware that they were being observed and their practice assessed. Any such bias was likely to have been in the direction of promoting a more thorough or accurate (according to current guidelines) clinical case consultation, although possible anxieties associated with the knowledge that one was being assessed may have negatively impacted on clinical performance in some cases. In health centre settings this source of potential bias was hopefully minimized given the duration of the observations (five days), although it cannot be discounted completely. The reported percentage of ‘correct’ anti-malarial prescriptions may be an overestimate as information on the severity of illness (uncomplicated vs. severe malaria) was not available. However, 96.1% (423/44) of patients included in this analysis were sent home at the conclusion of their clinical consultation suggesting uncomplicated malaria was the most likely diagnosis. Similarly, the assessment of whether an anti-malarial prescription was provided in the correct dosage (or not) was based on patient age as opposed to weight. Finally, the sample size employed in the regression analysis presented in Table 4 was low and the reported findings should be considered highly tentative.

## Conclusion

The findings presented in this paper strongly indicate that the new PNG national malaria treatment protocol will require a substantial change in clinical practice if it is to be correctly implemented and adhered to. Areas that will require the most change include the shift from presumptive to RDT/microscopy confirmed diagnosis, prescribing (or rather non-prescribing) of anti-malarials to patients who test negative for malaria infection, and the provision of thorough treatment counselling. The successful introduction and maintenance of the proposed changes to clinical practice, therefore, will likely necessitate a comprehensive clinician support programme, possibly inclusive of ‘booster’ training opportunities and regular clinical supervision. The introduction of a revised malaria treatment protocol also presents as an excellent opportunity to identify effective health worker support mechanisms via sound research methodology.

## Competing interests

The authors declare that they have no competing interests

## Authors’ contributions

JP coordinated data collection, conducted the data analysis and drafted the manuscript. MWH, PMS, IM designed the study and critically revised the manuscript. All authors’ read and approved the final manuscript.
